# Supplementary material for: Hepatoprotective Effect of Silymarin Herb in Prevention of Liver Dysfunction Using Pig as Animal Model
Source: Nutrients. 2025 Oct 18;17(20):3278. doi: 10.3390/nu17203278 (PMC12566644; doi:10.3390/nu17203278)
Supplement: Supplementary file 1 [file nutrients-17-03278-s001.zip › Figure S1.pdf]

SUPPLEMENTARY MATERIAL

Figure S1 Molecular mechanisms underlying silymarin's anti-inflammatory, antioxidant, and hepatoprotective effects.

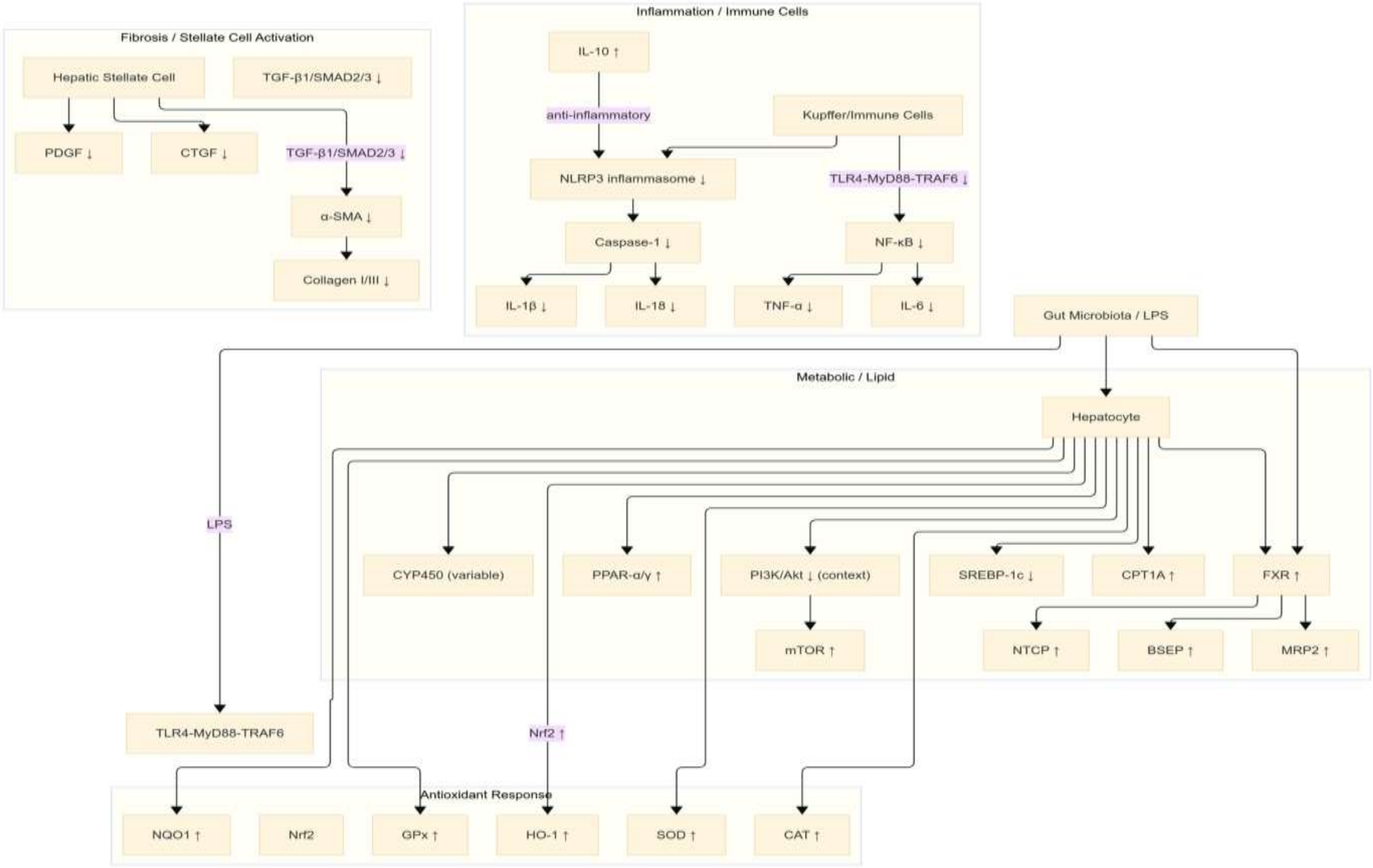

Figure Legend:

Silymarin acts at several points along the pathways that drive liver injury and repair. In stellate cells, it reduces fibrogenesis by slowing PDGF-driven proliferation, blunting TGF-β1/SMAD signalling, and lowering the expression of α-SMA and collagen, which together limit scar formation. In resident immune cells such as Kupffer cells, it tempers inflammation by blocking activation of the NLRP3 inflammasome and caspase-1, leading to reduced release of IL-1β, TNF-α, and IL-6, while supporting anti-inflammatory signalling through IL-10. At the metabolic level, silymarin dampens LPS–TLR4–MyD88 signalling and CYP2E1-derived oxidative stress, and it shifts lipid handling by modulating PPAR-γ, PI3K/AKT, and SREBP-1c. It also improves bile acid regulation by downregulating NTCP while enhancing FXR, BSEP, and MRP2 activity, thereby protecting hepatocytes against cholestatic stress. On the antioxidant front, it boosts Nrf2 activity and the downstream enzymes HO-1, NQO1, SOD, and catalase, strengthening the cell's defences against oxidative damage. Finally, its effects extend to the gut–liver axis, where it counteracts endotoxin-driven inflammation linked to microbial imbalance. Taken together, these overlapping actions explain how silymarin can simultaneously reduce fibrosis, suppress inflammation, restore metabolic balance, and reinforce antioxidant capacity in chronic liver disease.
