# Supplementary material for: Hepatoprotective Effect of Silymarin Herb in Prevention of Liver Dysfunction Using Pig as Animal Model
Source: Nutrients. 2025 Oct 18;17(20):3278. doi: 10.3390/nu17203278 (PMC12566644; doi:10.3390/nu17203278)
Supplement: Supplementary file 1 [file nutrients-17-03278-s001.zip › Table S1.pdf]

SUPPLEMENTARY MATERIAL

Table S1. Pharmacological properties and mechanisms of silymarin reported across experimental and clinical studies.

Study	Model	Intervention	Primary endpoints	Main findings	Mechanism/pathway	Relevance to pig/human	Citations
Wang et al., 2024	C57BL/6J mice with diet-induced NAFLD (8 wks induction + 16 wks intervention). Groups: control diet, high-fat diet, high-fat + silymarin, high-fat + polyherbal (silymarin, salvianolic acid B, puerarin).	Silymarin or polyherbal combo; oral supplementation for 16 weeks.	Liver histology (steatosis, function), serum lipids, untargeted metabolomics, 16S rRNA sequencing; fecal microbiome transplantation (FMT) validation.	↓ hepatic steatosis, ↑ liver function; ↑ beneficial gut microbes (*Akkermansia*, *Blautia*); ↓ harmful microbes (*Clostridium*, *Bacteroides*). Improved bile acid composition, reduced hepatic lipid accumulation.	Gut–liver axis modulation via microbiota reshaping; inhibition of secondary bile acid synthesis; improved bile acid–lipid metabolic signalling.	Highlights microbiota-dependent mechanism of silymarin; gut-liver axis conserved in pigs/humans, suggesting translational potential for NAFLD interventions.	[21]
Handu et al., 2025	Adults with MASLD/NASH; scoping review of clinical studies (2000–2023).	Bioactive-based supplementation including curcumin, silymarin, resveratrol, coffee, green tea, berberine, and others.	Clinical outcomes related to hepatic health, weight, body composition, glycemic control, blood lipids, and adverse events.	Screening of 4572 records yielded 131 primary studies and 49 systematic reviews. Most frequently studied compounds were curcumin (25), silymarin (17), resveratrol (10), coffee (7), green tea (5), and berberine (5). Outcome heterogeneity was substantial, with mixed but generally positive results. Silymarin demonstrated consistent hepatoprotective effects across included trials.	Clinical-level evidence for silymarin centers on antioxidant, anti-inflammatory, and lipid-modulating effects, though heterogeneity prevents firm mechanistic conclusions.	Positions silymarin among the most evidence-supported nutraceuticals for MASLD. Human data directly reinforce translational validity for animal models.	[96]
Scafuri et al., 2025	Systematic review of 45 randomized controlled trials in breast cancer patients.	Various dietary supplements (vitamins, minerals, herbal compounds, oral formulations), including silymarin.	Clinical outcomes and quality of life indices.	Supplements such as vitamin D, omega-3 fatty acids, beta-glucan, amino acids, and certain herbals (including silymarin) improved quality of life. Disease-specific outcomes were variable. Silymarin consistently showed hepatoprotective effects, supporting safe use in cancer patients.	Likely through antioxidant, anti-inflammatory, and hepatoprotective properties, aiding supportive care during cancer therapy.	Demonstrates safety and hepatoprotective benefits of silymarin in oncology patients, highlighting its utility as an adjunct therapy in supportive care settings.	[97]

Reguero et al., 2024	High-fat diet–induced obesity in mice.	Silymarin-enriched milk thistle extract; oral supplementation.	Energy expenditure, cold-exposure thermogenesis, insulin resistance, inflammatory markers in adipose tissue, mitochondrial activity, and gene expression.	Treatment increased systemic energy expenditure and preserved body temperature during cold stress. Improved insulin sensitivity and reduced inflammatory markers in white adipose tissue. Enhanced expression of thermogenic genes and mitochondrial function.	Activation of thermogenesis, improved mitochondrial activity, and reduction of meta-inflammation.	Suggests a role for silymarin in managing obesity-related metabolic dysfunction, which is strongly linked to fatty liver disease. Provides evidence for silymarin as a nutraceutical bridging metabolic and hepatic health.	[25]
Ranneh et al., 2024	Human NAFLD patients; systematic review of randomized controlled trials.	Polyphenol supplementation, including turmeric, curcumin, resveratrol, silymarin, hesperidin, naringenin, and green tea extract.	Liver enzymes (ALT, AST), inflammatory cytokines, lipid profiles, insulin resistance, and NAFLD scores.	Analysis of 29 RCTs involving 1,840 patients showed that turmeric/curcumin consistently reduced liver enzymes, inflammatory cytokines, lipid profiles, insulin resistance, and NAFLD scores. Most silymarin studies demonstrated reductions in liver enzymes and lipid parameters. Hesperidin, naringenin, and green tea extract improved inflammatory markers and hepatic function, though evidence was less consistent.	Antioxidant, anti-inflammatory, and metabolic modulation; silymarin particularly effective in enzyme and lipid regulation.	Provides robust clinical evidence that silymarin, alongside other polyphenols, improves NAFLD outcomes. Supports translational consistency from animal models to human patients.	[98]
Santamarina et al., 2024	Overweight adults (n=22; BMI 18.5–34.9); 180-day supplementation.	Nutraceutical formulation (prebiotics + minerals + silymarin; LL1 capsule).	Gut microbiota composition (16S rRNA sequencing), inflammatory cytokine levels, anthropometric parameters, mood, and sleep quality (questionnaires).	Supplementation significantly reshaped gut microbiota composition (↑ butyrate-producing bacteria; ↑ Bifidobacterium and Ruminococcus). Improvements observed in anthropometric measures, inflammatory cytokines, mood perception, and sleep quality.	Gut–brain axis modulation via microbiota-driven shifts and reduction in systemic inflammation.	Clinical demonstration of silymarin's indirect role in gut–brain modulation. Suggests broader systemic effects beyond hepatoprotection, though findings are preliminary and require larger trials.	[99]
Vrentzos et al., 2025	Narrative systematic review of clinical interventions for MASLD.	Nutritional strategies (Mediterranean diet, weight loss) and nutraceuticals (polyphenols,	Reduction in liver fat, inflammation, and fibrosis; improvement in clinical metabolic parameters.	Mediterranean diet was identified as the most effective dietary intervention. Weight loss >5% reduced liver fat; 7–10% reduced inflammation; ≥10% improved fibrosis.	Combined dietary and nutraceutical modulation of oxidative stress, lipid metabolism, and inflammatory signaling.	Positions silymarin within a broader nutraceutical framework for MASLD, reinforcing the importance of integrative strategies with lifestyle changes.	[100]

		omega-3 fatty acids, vitamin D, others).		Polyphenols, omega-3 fatty acids, and vitamin D provided additional benefits. Lifestyle modification remains the cornerstone of treatment, but nutraceuticals can enhance long-term outcomes.			
Bosco et al., 2024	Human pilot trial (n=20 wine-drinking subjects).	Combination supplement of silymarin, pyrroloquinoline quinone sodium salt (PQQ), and myricetin (Si.Pi.Mi.); randomized controlled, single-blind design.	Blood ethanol and ethyl glucuronide levels; oxidative stress biomarkers (ROS, total antioxidant capacity, CoQ10, thiols, 8-isoprostane, NO metabolites).	Si.Pi.Mi. supplementation reduced circulating ethanol by ~33% after 120 minutes. Total antioxidant capacity increased (+9–12%), while oxidative stress marker 8-isoprostane decreased (−22–27%).	Combined antioxidant and metabolic support; improved ethanol clearance and redox balance.	Provides preliminary clinical evidence that silymarin, in combination with other antioxidants, may enhance alcohol metabolism and oxidative stress defense. Results are promising but limited by small sample size and require larger validation studies.	[101]
Cicero et al., 2018	Clinical trials and observational studies in NAFLD patients.	Nutraceuticals including silymarin, vitamin E, vitamin D, omega-3 fatty acids, berberine, curcumin, and resveratrol.	Anthropometric (weight, BMI), biochemical (liver enzymes, lipid profile, insulin sensitivity), and hemodynamic parameters.	Silymarin, vitamin E, vitamin D, and omega-3 fatty acids consistently improved NAFLD-related parameters. Curcumin and berberine primarily improved liver enzyme activity. Clinical benefits required medium- to long-term supplementation.	Combination of antioxidant, anti-inflammatory, and metabolic effects; silymarin noted for its favorable safety profile.	Highlights the importance of long-term nutraceutical supplementation (particularly silymarin) in NAFLD management. Emphasizes translational consistency and safety in chronic disease models.	[102]
Gillessen and Schmidt, 2020	Clinical and preclinical (human trials in ALD, NAFLD, cirrhosis; cell/animal data)	Silymarin (Eurosil® 85 or comparable standardized extracts)	Liver enzymes (ALT/AST), liver-related mortality, glycemic parameters, clinical liver outcomes	Silymarin reduces oxidative stress, improves liver enzyme profiles, associated with reduced liver-related deaths in pooled cirrhosis trials; improved glycemic control in diabetic cirrhotics; well tolerated	Free-radical scavenging; modulation of enzymes involved in fibrosis and cytotoxicity; antioxidant, antifibrotic and membrane-stabilizing activity	Human: strong clinical relevance; widely-used standardized formulation. Pig: pathways conserved (useful for preclinical PK/PD and hepatoprotection models)	[103]
M. Li et al., 2021	Clinical literature review summarizing DILI therapies	Silymarin listed among hepatoprotective agents (others: NAC, glutathione,	Resolution of DILI, time to recovery, liver enzymes, progression prevention	Silymarin appears as a hepatoprotective option in DILI; evidence varies in quality but indicates benefit in certain etiologies	Antioxidant (ROS reduction, GSH enhancement), membrane stabilization, anti-inflammatory	Human: clinical practice relevance for DILI management; Pig: translational in preclinical DILI models	[104]

		glycyrrhizin, PPC, bicyclol)					
Wadhwa et al., 2022	Preclinical (cells, animals) and clinical data synthesis	Silymarin mixture (silybins, silychristin, silydianin, taxifolin)	Multiple organ-protective endpoints (hepatoprotection, neuro/cardio etc.), inflammatory/apoptotic markers	Silymarin shows multi-system benefits (hepatoprotective, antioxidant, anti-inflammatory, anti-apoptotic, metabolic effects) across models	Modulates MAPK, mTOR, Akt, β-catenin; suppresses apoptotic and inflammatory gene expression; antioxidant pathways	Human: mechanistic support for clinical findings; Pig: conserved molecular pathways support translational studies	[105]
Rahimi-Dehkordi et al., 2024	Clinical: pediatric β-thalassemia trials (systematic review)	Silymarin adjunctive therapy	Hematological parameters, oxidative stress markers, iron metabolism, liver enzymes	Silymarin reduces ROS, increases GSH, attenuates inflammation, reduces iron overload markers, improves liver enzymes and RBC parameters; safe in studied doses	Antioxidant (↑GSH), anti-inflammatory (↓TNF-α, TGF-β1, ILs), iron-modulation/chelation	Human (pediatrics): positive adjunctive effects; Pig: translational potential for iron-overload models	[106]
Nehmi-Filho et al., 2022	Human double-blind RCT in sedentary adults (BMI ≤34.9)	Nutraceutical supplements with or without silymarin (combined with β-glucan, prebiotics, minerals)	Anthropometrics (waist circumference, WHR), liver enzymes, endocrine/metabolic markers	Supplementation (including silymarin) reduced waist circumference metrics, improved AST/ALT ratio and some endocrine markers (cortisol, TSH) vs baseline	Antioxidant and metabolic modulation; likely effects on inflammation and liver biomarkers	Human: supports potential metabolic benefits when used with other nutraceuticals; Pig: may guide combined-supplement testing in metabolic models	[107]
Abenavoli et al., 2018	Preclinical (in vitro/animal) and clinical review	Silymarin (mainly silybin)	Liver function tests, oxidative stress, fibrosis markers	Silymarin has antioxidant, anti-inflammatory, antifibrotic, immunomodulatory, and hepatoregenerative properties; clinical potential in ALD, NAFLD, viral hepatitis, DILI	Scavenging ROS, ↑GSH, inhibition of stellate-cell activation, cytokine suppression	Human: summarizes clinical potential; Pig: useful for preclinical pharmacology and dosing studies	[15]
Abenavoli et al., 2010	Review of animal and human studies	Silymarin (silybin predominant)	Liver injury prevention/treatment endpoints	Silymarin reduces liver injury in many toxin/disease models and has clinical uses in ALD, viral and toxin-induced injuries	Antioxidant, antifibrotic, membrane-stabilizing; possible toxin-receptor blockade	Human: clinically relevant; Pig: translationally useful for hepatoprotection and regeneration	[108]
Yang et al., 2022	Systematic review & meta-analysis of RCTs in NAFLD (2,173 participants)	Polyphenol interventions (curcumin, resveratrol, catechin, silymarin etc.)	ALT/AST, hepatic fat, liver stiffness, metabolic markers	Silymarin improved ALT/AST and reduced hepatic fat and liver stiffness; overall polyphenol results mixed but select compounds (incl. silymarin) showed benefit	Antioxidant, anti-inflammatory, insulin-sensitivity improvement	Human: RCT-based support for silymarin in NAFLD; Pig: NAFLD pig models translatable	[109]
S. Li et al., 2024	Systematic review & meta-analysis (26 RCTs, 2,375 pts)	Clinical RCTs with silymarin	ALT/AST, lipid profile, FI/HOMA-IR, fatty liver index, histology	Silymarin ↓ TC, TG, LDL-C; ↑ HDL-C; ↓ ALT/AST; improved	Regulates energy/lipid metabolism, antioxidant	Human: strong pooled evidence for metabolic and hepatic benefit; Pig:	[111]

				FI and fatty liver indices; improved histological steatosis	and anti-inflammatory effects, antifibrotic	mechanistic endpoints translatable	
Zhao et al., 2024	Preclinical (cell) and clinical reviews	Silymarin (various formulations)	Inflammatory markers (NF-κB, MAPK), clinical inflammation endpoints	Silymarin reduces pro-inflammatory factor secretion and modulates clinical inflammation; safe	Inhibits NF-κB and MAPK pathways, activates antioxidant responses, lowers cytokines	Human: supports use in inflammatory liver/autoimmune contexts; Pig: pathways conserved for translational studies	[112]
Wah Kheong et al., 2017	RCT: adults with biopsy-proven NASH (NAS ≥4)	Silymarin 700 mg TID for 48 weeks vs placebo	Primary: ≥30% reduction in NAS; Secondary: fibrosis, steatosis, ballooning, enzymes, liver stiffness	No significant difference in primary NAS endpoint; significant reductions in fibrosis (histology 22.4% vs 6%) and liver stiffness measures; safe	Antioxidant, anti-inflammatory, antifibrotic activities	Human: direct clinical RCT evidence suggesting fibrosis benefit; Pig: model for fibrosis testing/translational dosing	[113]
Jin et al., 2024	RCT: MASLD patients (n≈83), 24 weeks	Silymarin 103.2 mg/day vs placebo	Liver stiffness (FibroScan), CAP (steatosis), biochemical markers, gut microbiota composition	Reduced liver stiffness (LSM), ↓ GGT and ApoB; no change in CAP; increased gut microbiota diversity and Oscillospiraceae enrichment	Gut microbiota modulation + antioxidant/anti-inflammatory effects	Human: links microbiome shifts to liver stiffness improvement; Pig: gut-liver axis models translatable	[110]
Zarif-Yeganeh & Rastegarpanah, 2019	Short review: animal models + limited clinical data	Silymarin supplement	Oxidative stress markers, sperm/oocyte parameters, fertility outcomes	Protective antioxidant effects on gametes; improved fertility in animal studies; potential clinical benefit but need RCTs	Free-radical scavenging; NF-κB inhibition; possible phytoestrogenic activity	Human: potential application in infertility; Pig: reproductive physiology allows translational studies	[114]

Yan et al., 2020	Review across preclinical & clinical THM studies	Silymarin among promising THM candidates	NAFLD endpoints (steatosis, inflammation, fibrosis)	Silymarin highlighted as anti-NAFLD candidate; supports multiple-organ "multiple-hits" model	Antioxidant, anti-inflammatory, metabolic regulation across organs	Human: supports clinical development; Pig: NAFLD pig models recommended for preclinical validation	[115]
Pradhan & Girish, 2006	Review of experimental pharmacology → clinical medicine	Silymarin (mixture of flavonolignans)	Hepatoprotection endpoints, toxin models, liver recovery	Strong hepatoprotection across toxic models; clinical applications in ALD, cirrhosis, toxin-induced injury; safe and affordable	Antioxidant, anti-lipid peroxidative, antifibrotic, anti-inflammatory, immunomodulatory, membrane stabilization, regenerative	Human: broad hepatoprotective relevance; Pig: translational for hepatotoxicity/regeneration research	[116]
Xie et al., 2019	In vitro transporter systems and animal PK models	Silybin / silymarin	PK/transport endpoints, transporter interactions	Major efflux via MRP2 and BCRP; inhibitors can enhance absorption; silymarin safe with limited clinically relevant DDIs	Efflux transporter modulation (MRP2 / BCRP); Phase II conjugation (glucuronidation/sulfation)	Human: important for dosing & DDI considerations; Pig: transporter conservation supports translational PK studies	[117]

Saller et al., 2008	Clinical double-/single-blind trials reviewed	Silymarin clinical trials	AST/ALT, mortality, clinical endpoints in cirrhosis and toxic hepatitis	AST reduced in alcoholic liver disease; liver-related mortality reduced in cirrhosis; little effect in viral hepatitis	Antioxidant, hepatoprotective, potential toxin blockade	Human: supports silymarin as supportive therapy in alcoholic cirrhosis and Amanita poisoning; Pig: translational for toxic injury models	[118]
Milosević et al., 2014	Clinical NAFLD studies and supportive preclinical data	Silymarin (lipophilic extract)	Steatosis grade, ballooning, fibrosis, transaminases, insulin sensitivity	Silymarin reduces steatosis, ballooning, fibrosis and ALT/AST; can improve insulin sensitivity; fewer side effects vs some pharmacotherapies	Antioxidant, antifibrotic, metabolic modulation, anti-inflammatory	Human: supports silymarin as an effective NAFLD agent; Pig: NAFLD pig models applicable	[119]
Mohtashaminia et al., 2022	Systematic review & meta-analysis (5 RCTs, n=549)	Berberine + silymarin supplements	ALT, AST outcomes	No statistically significant changes in ALT or AST in pooled trials	No clear hepatoprotective signal for combined product at studied doses; reasons may include dose/formulation differences or study quality	Human: indicates neutral effect of BBR+silymarin on enzymes; Pig: could study PK interactions	[120]

Mastron et al., 2015	Preclinical HCC cell and animal models; limited clinical data	Silymarin flavonolignans	Tumor growth, apoptosis, oxidative stress, inflammatory markers	Silymarin shows chemopreventive/therapeutic potential (inhibits tumor growth, induces apoptosis, reduces oxidative stress)	Antioxidant (ROS scavenging), NF-κB inhibition, pro-apoptotic signaling	Human: promising translational potential for HCC chemoprevention; Pig: useful for translational oncology/fibrosis→HCC progression models	[121]

References: 15,21,25,96-121

21. Wang, X.; Jin, Y.; Di, C.; Zeng, Y.; Zhou, Y.; Chen, Y.; Pan, Z.; Li, Z.; Ling, W. Supplementation of Silymarin Alone or in Combination with Salvianolic Acids B and Puerarin Regulates Gut Microbiota and Its Metabolism to Improve High-Fat Diet-Induced NAFLD in Mice. *Nutrients* 2024, *16*, 1169, doi:10.3390/nu16081169.

96. Handu, D.; Stote, K.; Piemonte, T. Evaluating Bioactive-Substance-Based Interventions for Adults with MASLD: Results from a Systematic Scoping Review. *Nutrients* 2025, *17*, 453, doi:10.3390/nu17030453.

97. Scafuri, L.; Buonerba, C.; Strianese, O.; de Azambuja, E.; Palleschi, M.; Riccio, V.; Marotta, V.; Scocca, C.; Riccio, G.; Errico, C.; et al. Impact of Dietary Supplements on Clinical Outcomes and Quality of Life in Patients with Breast Cancer: A Systematic Review. *Nutrients* 2025, *17*, 981, doi:10.3390/nu17060981.

25. Reguero, M.; Reglero, G.; Quintela, J.C.; Ramos-Ruiz, R.; Ramírez de Molina, A.; Gómez de Cedrón, M. Silymarin-Enriched Extract from Milk Thistle Activates Thermogenesis in a Preclinical Model of High-Fat-Diet-Induced Obesity to Relieve Systemic Meta-Inflammation. *Nutrients* 2024, *16*, 4166, doi:10.3390/nu16234166.

98. Ranneh, Y.; Bedir, A.S.; Abu-Elsaoud, A.M.; Al Raish, S. Polyphenol Intervention Ameliorates Non-Alcoholic Fatty Liver Disease: An Updated Comprehensive Systematic Review. *Nutrients* 2024, *16*, doi:10.3390/nu16234150.

99. Santamarina, A.B.; Nehmi Filho, V.; Freitas, J.A. de; Franco, L.A.M.; Fonseca, J.V.; Martins, R.C.; Turri, J.A.O.; Silva, B.F.R.B. da; Gusmão, A.F.; Olivieri, E.H.R.; et al. Nutraceutical Capsules LL1 and Silymarin Supplementation Act on Mood and Sleep Quality Perception by Microbiota–Gut–Brain Axis: A Pilot Clinical Study. *Nutrients* 2024, *16*, 3049, doi:10.3390/nu16183049.

100. Vrentzos, E.; Pavlidis, G.; Korakas, E.; Kountouri, A.; Pliouta, L.; Dimitriadis, G.D.; Lambadiari, V. Nutraceutical Strategies for Metabolic Dysfunction-Associated Steatotic Liver Disease (MASLD): A Path to Liver Health. *Nutrients* 2025, *17*, 1657, doi:10.3390/nu17101657.

101. Bosco, G.; Vezzoli, A.; Brizzolari, A.; Paganini, M.; Giacon, T.A.; Savini, F.; Gussoni, M.; Montorsi, M.; Dellanoce, C.; Mrakic-Sposta, S. Consumption of Sylimarin, Pyrroloquinoline Quinone Sodium Salt and Myricetin: Effects on Alcohol Levels and Markers of Oxidative Stress—A Pilot Study. *Nutrients* 2024, *16*, 2965, doi:10.3390/nu16172965.
102. Cicero, A.F.G.; Colletti, A.; Bellentani, S. Nutraceutical Approach to Non-Alcoholic Fatty Liver Disease (NAFLD): The Available Clinical Evidence. *Nutrients* 2018, *10*, 1153, doi:10.3390/nu10091153.
103. Gillessen, A., & Schmidt, H. H.-J. (2020). Silymarin as supportive treatment in liver diseases: A narrative review. *Advances in Therapy*, *37*(4), 1279–1301. https://doi.org/10.1007/s12325-020-01251-y
104. Li, M., Luo, Q., Tao, Y., Sun, X., & Liu, C. (2021). Pharmacotherapies for drug-induced liver injury: A current literature review. *Frontiers in Pharmacology*, *12*, 806249. https://doi.org/10.3389/fphar.2021.806249
105. Wadhwa, K., Pahwa, R., Kumar, M., Kumar, S., Sharma, P. C., Singh, G., Verma, R., Mittal, V., Singh, I., Kaushik, D., & Jeandet, P. (2022). Mechanistic insights into the pharmacological significance of silymarin. *Molecules (Basel, Switzerland)*, *27*(16), 5327. https://doi.org/10.3390/molecules27165327
106. Rahimi-Dehkordi, N., Heidari-Soureshjani, S., & Sherwin, C. M. T. (2024). The effects and safety of Silymarin on β-thalassemia in children and adolescents: A systematic review based on clinical trial studies. *Reviews on Recent Clinical Trials*, *19*(4), 242–255. https://doi.org/10.2174/0115748871305325240511122602
107. Nehmi-Filho, V., Santamarina, A. B., de Freitas, J. A., Trarbach, E. B., de Oliveira, D. R., Palace-Berl, F., de Souza, E., de Miranda, D. A., Escamilla-Garcia, A., Otoch, J. P., & Pessoa, A. F. M. (2022). Novel nutraceutical supplements with yeast β-glucan, prebiotics, minerals, and Silybum marianum (silymarin) ameliorate obesity-related metabolic and clinical parameters: A double-blind randomized trial. *Frontiers in Endocrinology*, *1 3*, 1089938. https://doi.org/10.3389/fendo.2022.1089938
15. Abenavoli, L., Izzo, A. A., Milić, N., Cicala, C., Santini, A., & Capasso, R. (2018). Milk thistle (Silybum marianum): A concise overview on its chemistry, pharmacological, and nutraceutical uses in liver diseases. *Phytotherapy Research: PTR*, *32*(11), 2202–2213. https://doi.org/10.1002/ptr.6171
108. Abenavoli, L., Capasso, R., Milic, N., & Capasso, F. (2010). Milk thistle in liver diseases: past, present, future. *Phytotherapy Research: PTR*, *24*(10), 1423–1432. https://doi.org/10.1002/ptr.3207
109. Yang, K., Chen, J., Zhang, T., Yuan, X., Ge, A., Wang, S., Xu, H., Zeng, L., & Ge, J. (2022). Efficacy and safety of dietary polyphenol supplementation in the treatment of non-alcoholic fatty liver disease: A systematic review and meta-analysis. *Frontiers in Immunology*, *13*, 949746. https://doi.org/10.3389/fimmu.2022.949746
110. Jin, Y., Wang, X., Chen, K., Chen, Y., Zhou, L., Zeng, Y., Zhou, Y., Pan, Z., Wang, D., Li, Z., Liang, Y., Ling, W., & Li, D. (2024). Silymarin decreases liver stiffness associated with gut microbiota in patients with metabolic dysfunction-associated steatotic liver disease: a randomized, double-blind, placebo-controlled trial. *Lipids in Health and Disease*, *23*(1), 239. https://doi.org/10.1186/s12944-024-02220-y
111. Li, S., Duan, F., Li, S., & Lu, B. (2024). Administration of silymarin in NAFLD/NASH: A systematic review and meta-analysis. *Annals of Hepatology*, *29*(2), 101174. https://doi.org/10.1016/j.aohep.2023.101174
112. Zhao, Y., Zhou, Y., Gong, T., Liu, Z., Yang, W., Xiong, Y., Xiao, D., Cifuentes, A., Ibáñez, E., & Lu, W. (2024). The clinical anti-inflammatory effects and underlying mechanisms of silymarin. *IScience*, *27*(11), 111109. https://doi.org/10.1016/j.isci.2024.111109

113. Wah Kheong, C., Nik Mustapha, N. R., & Mahadeva, S. (2017). A randomized trial of silymarin for the treatment of nonalcoholic steatohepatitis. *Clinical Gastroenterology and Hepatology: The Official Clinical Practice Journal of the American Gastroenterological Association*, *15*(12), 1940-1949.e8. https://doi.org/10.1016/j.cgh.2017.04.016
114. Zarif-Yeganeh, M., & Rastegarpanah, M. (2019). Clinical role of silymarin in oxidative stress and infertility: A short review for pharmacy practitioners. *Journal of Research in Pharmacy Practice*, *8*(4), 181–188. https://doi.org/10.4103/jrpp.JRPP_18_100
115. Yan, T., Yan, N., Wang, P., Xia, Y., Hao, H., Wang, G., & Gonzalez, F. J. (2020). Herbal drug discovery for the treatment of nonalcoholic fatty liver disease. *Acta Pharmaceutica Sinica. B*, *10*(1), 3–18. https://doi.org/10.1016/j.apsb.2019.11.017
116. Pradhan, S. C., & Girish, C. (2006). Hepatoprotective herbal drug, silymarin from experimental pharmacology to clinical medicine. *The Indian Journal of Medical Research*, *124*(5), 491–504. https://www.ncbi.nlm.nih.gov/pubmed/17213517
117. Xie, Y., Zhang, D., Zhang, J., & Yuan, J. (2019). Metabolism, transport and drug-drug interactions of silymarin. *Molecules (Basel, Switzerland)*, *24*(20), 3693. https://doi.org/10.3390/molecules24203693
118. Saller, R., Brignoli, R., Melzer, J., & Meier, R. (2008). An updated systematic review with meta-analysis for the clinical evidence of silymarin. *Forschende Komplementarmedizin (2006)*, *15*(1), 9–20. https://doi.org/10.1159/000113648
119. Milosević, N., Milanović, M., Abenavoli, L., & Milić, N. (2014). Phytotherapy and NAFLD--from goals and challenges to clinical practice. *Reviews on Recent Clinical Trials*, *9*(3), 195–203. https://doi.org/10.2174/1574887109666141216110337
120. Mohtashaminia, F., Amini, M. R., Sheikhhossein, F., Djafarian, K., & Shab-Bidar, S. (2022). Effects berberine-silymarin on liver enzymes: A systematic review and meta-analysis of randomized controlled trials. *Clinical Nutrition ESPEN*, *49*, 181–186. https://doi.org/10.1016/j.clnesp.2022.01.037
121. Mastron, J. K., Siveen, K. S., Sethi, G., & Bishayee, A. (2015). Silymarin and hepatocellular carcinoma: a systematic, comprehensive, and critical review. *Anti-Cancer Drugs*, *26*(5), 475–486. https://doi.org/10.1097/CAD.0000000000000211
